# Role of cytokinins in seed development in pulses and oilseed crops: Current status and future perspective

**DOI:** 10.3389/fgene.2022.940660

**Published:** 2022-10-12

**Authors:** Sandhya Sharma, Parampreet Kaur, Kishor Gaikwad

**Affiliations:** ^1^ National Institute for Plant Biotechnology, Indian Council of Agricultural Research, New Delhi, India; ^2^ Punjab Agricultural University, Ludhiana, Punjab, India

**Keywords:** cytokinin, yield contributing traits, pulses and oilseeds, seed development, CKX, cytokinin oxidase/dehydrogenase

## Abstract

Cytokinins constitutes a vital group of plant hormones regulating several developmental processes, including growth and cell division, and have a strong influence on grain yield. Chemically, they are the derivatives of adenine and are the most complex and diverse group of hormones affecting plant physiology. In this review, we have provided a molecular understanding of the role of cytokinins in developing seeds, with special emphasis on pulses and oilseed crops. The importance of cytokinin-responsive genes including cytokinin oxidases and dehydrogenases (*CKX*), isopentenyl transferase (*IPT*), and cytokinin-mediated genetic regulation of seed size are described in detail. In addition, cytokinin expression in germinating seeds, its biosynthesis, source-sink dynamics, cytokinin signaling, and spatial expression of cytokinin family genes in oilseeds and pulses have been discussed in context to its impact on increasing economy yields. Recently, it has been shown that manipulation of the cytokinin-responsive genes by mutation, RNA interference, or genome editing has a significant effect on seed number and/or weight in several crops. Nevertheless, the usage of cytokinins in improving crop quality and yield remains significantly underutilized. This is primarily due to the multigene control of cytokinin expression. The information summarized in this review will help the researchers in innovating newer and more efficient ways of manipulating cytokinin expression including *CKX* genes with the aim to improve crop production, specifically of pulses and oilseed crops.

## Introduction

The importance of oilseeds and pulses in the human diet cannot be overstated. Wherein oilseeds comprise high-energy food with double the amount of energy as carbohydrate and protein (AGRIS FAO, 2022), pulses fulfill protein demands of the majority of people, and both the crop varieties are important for agriculture and livestock. Hormones constitute a pivotal component of regulatory mechanisms directing plant development and have been extensively studied with respect to various seed attributes.

In India, pulses and oilseeds are vital components of the food and nutritional security. India’s dietary habits are still predominantly vegetarian, and the country relies primarily on plant-based sources to achieve its daily protein and other nutritional needs. In addition, according to the FAO, pulses are an important part of a balanced diet. Pulses have been linked to lower risk factors for chronic disease. Apart from being an important aspect of human nutrition, pulses also play a key role in sustainable agriculture and climate change mitigation (Fao 2017; Ha et al., 2014; Jayalath et al., 2014; Kim et al., 2016; Sievenpiper et al., 2009; Viguiliouk et al., 2017; Calles, 2016; Foyer et al., 2016). Over the last 15 years, India has made significant success in increasing pulse production. In 2005–06, India’s total pulse production was 13.38 million metric tonnes (MT), which rose to 25.58 million MT in 2020–21. This represents a 91% increase, or a compound annual growth rate (CAGR) of 4.42%. Regarding oilseeds production, India grows roughly 15%–20% of the world’s total output, produces 6%–7% of vegetable oils, and consumes 9%–10% of all edible oils. Oilseeds are only second to food grains in terms of acreage, production, and economic worth.

**FIGURE 1:**
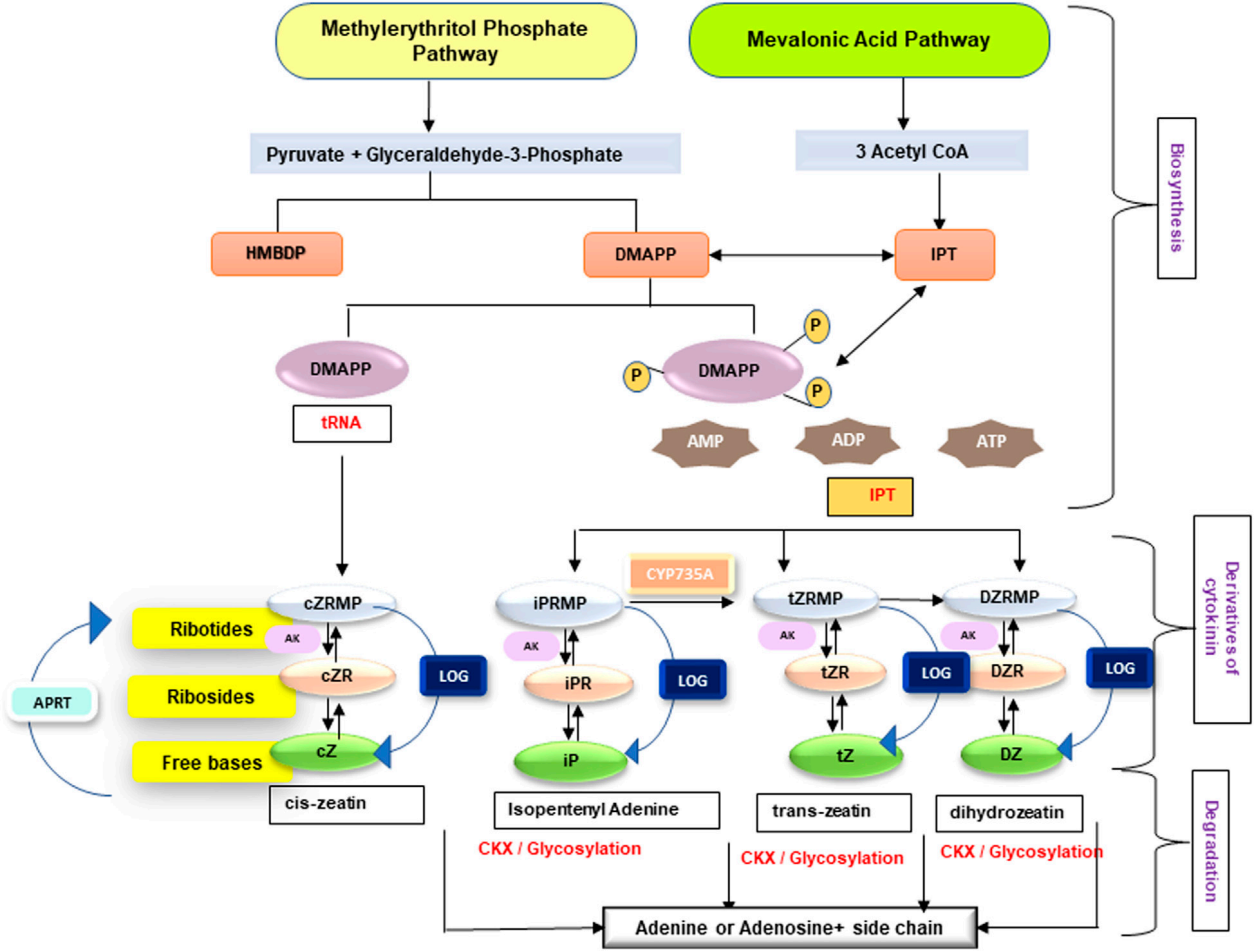
An overview on biosynthesis and enzymes of cytokinin homeostasis (iP, isopentenyladenine; DHZ, dihydrozeatin; cZ, cis-zeatin; tZ, trans-zeatin; DMAPP, dimethylallyl pyrophosphate; AMP, adenosine monophosphate) cytokinin biosynthetic pathways—mevalonic acid pathway (operates in cytosol and mitochondria) and methylerythritol pathway (operates in plastids). Enzymes involved are 1) IPT (isopentenyl transferase), which uses ATP, ADP, or ATP as acceptor and forms iPRTP (riboside 5′-diphosphate), iPRDP (riboside 5′-diphosphate), and iPRMP (riboside 5′-monophosphate); 2) CYP735A converts CK nucleotides in to tZ nucleotides; 3) AK (adenosine kinase) causes the phosphorylation of ribosides iPR, tZR, cZR, and DZR; 4) LOG (LONELY GUY) produces free bases from ribotides; 5) APRT (adenine phosphoribosyltransferase) catalyzes the conversion of CK bases to nucleotides; and 6) CKX (cytokinin oxidase/dehydrogenase) causes catabolism of free bases (iP, tZ, DZ, and cZ) to adenine or adenosine (for references, see Takei et al., 2004; Kudo et al., 2010; Sakakibara 2006; Kurakawa et al., 2007; Schoor et al., 2011).

Cytokinins are one of the most well studied plant hormones, exercising huge physiological and molecular impact throughout the life cycle of a plant. Cytokinins regulate several functions, such as root development, formation and maintenance of shoot meristem, organ formation, seed germination, seed and fruit development, senescence delay, and response to abiotic and biotic stress. An overview about cytokinins in terms of their biosynthesis, types, bioavailability, and storage forms is described in Figure 1. Cytokinin homeostasis is maintained through action of various enzymes involved in their activation, irreversible conjugation, and degradation. Cytokinins are present throughout the parts of higher plants, though abundantly in the tips of roots, apical meristem of the shoots, and the immature seeds. Majority of higher plants have more than a dozen cytokinins forms which are capable of interconversion. A plethora of previously undertaken studies (Jameson, et al., 1987; Werner and Schmülling, 2009; Kudo et al., 2010; Spichal and Spichal 2012; Schaller et al., 2014; Zwack and Rashotte, 2015) have provided a detailed and extensive overview of cytokinin.

Attempts have been made toward understanding the cytokinin-mediated molecular mechanism regulating important agronomic traits including plant height, plant density, date of flowering, number of primary and secondary branches, seed number per pod/silique, number of pod per plants, pod length, thousand seed weight, and seed size including seed length and seed width.

### Cytokinin biosynthetic pathways and its regulation

Naturally occurring cytokinins are derivatives of adenine with an aromatic or an isoprenoid side chain of isopentenyladenine [N6-(D2-isopentenyl) adenine] and hydroxylated either at *cis*- or *trans-*terminal position, thereby forming zeatin, named after its discovery in maize (Strnad 1997; Mok and Mok 2001). Among these, cytokinins with the r side chains are widespread in nature. Later in mid-1970s, the presence of benzyladenine was reported in pea, a leguminous plant (Gaudinova’ et al., 2005). Numerous researchers in the past have reported the dominance of *trans*-zeatin and isopentenyladenine derivatives in nature, whereas *cis*-isomer is present in very low concentration and has very little or no activity. In contrast to this, recent studies on phytohormones revealed the abundance of *cis*-isomers in several legume species (Emery et al., 1998, 2000; Quesnelle and Emery, 2007) and other plants such as rice (Takagi et al., 1985) and maize (Veach et al., 2003; Vyroubalova’ et al., 2009). Naturally occurring derivatives of cytokinin encompass N6-(2-isopentenyl) adenine (iP), *trans*-zeatin (tZ), *cis*-zeatin (cZ), and dihydrozeatin (DHZ), which are a part of isoprenoid cytokinins, whereas benzyladenine (BA) along with its hydroxylated derivatives ortho- and metatopolin (oT and mT) and their methoxy-derivatives are included under the aromatic cytokinins group. Theoretically, 26 molecular species of cytokinin have been discovered (Murai, 2014). In total, seven distinct modifications at positions including, N9-position ribosides in adenine mono-, di-, and tri-phosphate ribonucleotides; at N7- and N9-positions glucosides; and O-glucoside in the isopentenyl side chain have been observed so far (Mok and Mok 1994; 2004; Sakakibara 2006; Kojima et al., 2009).

Essentially, two pathways are involved in the biosynthesis of cytokinin precursors, that is, isopentenyladenine-dependent pathway (mevalonic acid pathway) in the cytosol and mitochondria and isopentenyladenine-independent pathway (methylerythritol pathway) in the plastids in order to form isopentenyl transferase (IPT) and dimethylallyl pyrophosphate (DMAPP) as precursors in cytokinin biosynthesis (Figure 1). Subsequently, isopentenyl transferase (IPT) catalyzes transfer of isopentenyl moiety from DMAPP or hydroxymethyl butenyldiphosphate (HMBDP) to either AMP, ADP, or ATP for the formation of biologically active cytokinins and constitutes the rate limiting step. Furthermore, zeatin-type cytokinins are produced by the hydroxylation of the isopentenyl side chain. Alternatively, the hydroxylated side chains can be inserted straight to the N6-position of adenine moiety leading to formation of adenylate IPT. The isopentenyladenine derivative thus produced is then converted to *trans*-zeatin adenine by an action of a root localized cytochrome P450 monooxygenases (Takei et al., 2004; Kiba et al., 2013), thereby limiting *trans-*zeatin synthesis to roots but readily transported to other plant parts *via* xylem. The interconversion between the *cis* and the *trans*-isomer of zeatin is mediated by the enzyme *cis*-*trans* zeatin isomerase. Macro-concentration of cytokinin in plants is controlled by action of IPT and CKX enzymes; however, conversion of cytokinin nucleotides to cytokinin bases is catalyzed by enzymes, that is, cytokinin phosphoribosyl hydrolase (LOG) (Kurakawa et al., 2007; Kuroha et al., 2009; Tokunaga et al., 2012) and reverse action, that is, conversion from cytokinin bases to nucleotides is catalyzed by adenine phosphoribosyl transferases (APRTs) enzymes (Zhang et al., 2013). All the genes involved in cytokinin homeostasis exists as multi-gene families, with the individual members being differentially expressed in space and time. For instance, 8 and 9 *IPT* cDNAs and genes, respectively, have been reported in *Arabidopsis* genome. Of these, seven *IPT* genes use adenine nucleotides as substrate for transfer reaction, except *IPT2*. In addition, three of the *IPT* genes are expressed in the plastids (*IPT1*, 5, and 8), whereas the rest are localized to the cytoplasm. Furthermore, each gene has different spatial expression profile, such as *IPT6* expresses in siliques, *IPT4* in immature seeds, and *IPT3* in phloem tissues.

### Role of kinases in cytokinin signaling

Cytokinins act by regulating the expression of several genes downstream of the signaling cascade. The signaling mechanism of cytokinins in plants is unique and is very similar to the bacterial two component system (Stock et al., 2000; Cheung and Hendrickson, 2010). It involves a transmembrane histidine kinase receptor which dimerizes on ligand binding followed by autophosphorylation of the receptor. This leads to recruitment of an intermediate histidine phosphotransfer protein (HP) which in turns causes phosphorylation of downstream proteins known as nuclear response regulators in the nucleus which then execute cytokinin action. They either regulate the expression of cytokinin response genes as transcription factors or activate different downstream proteins as protein kinases. A detailed characterization of these response regulators in *Arabidopsis*, which in conjugation, led to their classification in four different classes: type-A, type-B, type-C, and pseudo response regulators (PRRs). The type-B response regulators are DNA-binding transcription factors that promote the expression of cytokinin primary response genes.

Recent evidences suggest the presence of cytokinin response factors in *Arabidopsis* and in conjugation with response regulators mediate the expression of cytokinin-responsive genes. The mode of signaling involved is either paracrine (local signal in meristematic tissues) or distal signaling (for signaling of availability of nutrients). Furthermore, the selective transport of the two most common cytokinins-*trans*-zeatin (tz) and isopentenyladenine (iP) is mediated specifically by xylem and phloem, respectively. The histidine kinases involved in the cytokinin signaling cascade are the transmembrane receptors that comprises of an extracellular CHASE domain (cyclases/histidine kinases–associated sensory extracellular) on which the ligand binds leading to the dimerization and hence activation of the receptor (Inoue et al., 2001; Nishimura et al., 2004, Nishimura et al., 2004 C.; Higuchi et al., 2004). The conserved histidine kinase domain is essential for the activation through autophosphorylation. The receiver domain comprises of a conserved aspartate domain which plays a key role in the transfer of phosphate group from HK domain to the histidine phosphotransfer protein (HP) that further conveys the signal specifically to the type-B response regulator (Appleby et al., 1996; Schaller et al., 2011) in the nucleus. The type-B response regulators possess a DNA-binding domain, thereby acting as transcription factors. In contrast to this, type-A response regulators do not have a DNA-binding domain and their downstream proteins are still undetermined. Once activated, the type-B response regulators are able to activate all the cytokinin-responsive genes including the type-A response regulators, which in turn suppress cytokinin signaling, thereby providing a negative feedback loop during the signaling pathway (Pareek et al., 2006; Du et al., 2007; Pils and Heyl, 2009; Kieber and Schaller, 2014).

### Cytokinin-mediated genetic and epigenetic regulation of seed size

One of the key aspects of increasing crop productivity is the seed size. The major emphasis of crop production or improvement since time immemorial has been the selection of crops with bigger seed size (Song et al., 2007; Shomura et al., 2008; Fan et al., 2009). Seed size varies dramatically between species. The endosperm makes up the majority of the mature seed in monocots like rice and wheat. Majority of the dicots, for e.g., *Arabidopsis thaliana* and *Brassica napus* develop their endosperm rapidly at the initial stage; as a consequence, the embryo occupies the larger part of the developed mature seed. In flowering plants, the development of seeds is influenced by complex interactions between maternal tissues, embryo, and endosperm. It has been observed that the endosperm has a major role in regulating seed size. The endosperm exhibits more expeditive growth as compared to embryo during the early phase of seed development, and the seed volume also increases vis a vis the endosperm’s growth (Sundaresan, 2005). Several genes and transcription factors regulating growth of endosperm have also been reported to regulate the seed size in *Arabidopsis*. For example, *HAIKU1 (IKU1)*, *HAIKU2* (*IKU2*), and *MINISEED3* (*MINI3*) have been reported to function synergistically in the same genetic pathway to enhance the endosperm size and embryo development (Garcia et al., 2003; Luo et al., 2005; Zhou et al., 2009). Promoters for *MINI3* and *IKU2* also associate with Short Hypocotyl Under Blue 1 (*SHB1*) transcription factor (recruited by *WRKY10*) to promote endosperm development (Zhou et al., 2009). A number of reviews have detailed out the role of these genes in the genetic regulation of seed size (Sundaresan, 2005; Sun et al., 2010; Kesavan et al., 2013). In addition, several maternally derived factors have also been shown to regulate seed size in various plants (Jofuku et al., 2005; Li et al., 2008; Adamski et al., 2009; Ohto et al., 2009; Xia et al., 2013; Singh et al., 2017). Recent studies have highlighted the involvement of various transcription factors, G protein-coupled hormone signaling and ubiquitin-mediated pathway in maternal control of seed size in *Arabidopsis*. The cytokinin oxidase 2 (*CKX2*) gene produces a protein that destroys active cytokinin present in the cell in an irreversible manner. The IKU pathway controls seed size *via* regulating endosperm growth, and *CKX2* has been identified as a direct transcriptional target of the IKU system (Li et al., 2013). Overexpression of *CKX2* resulted in the recovery from the decrease in seed size phenotypes, indicating the involvement of *CKX2* in regulating seed size in a positive way. DNA methyltransferase 1 (*MET1*) (causes methylation of cytosine in CG) regulates *CKX2* as well as epigenetic maternal imprinting (Li et al., 2013). Membrane-bound cytokinin receptors are encoded by the arabidopsis histidine kinases (AHK) family, and *AHK2*, *AHK3*, and *CRE1* (cytokinin response 1)/*AHK4* are the three histidine kinases that bind cytokinin. Although the deletion of one or both of these receptors had no effect on seed size, *ahk1 ahk2*, and *ahk3* triple mutant seeds exhibited a 250% higher volume, with embryo cell number and size increasing by 15% and 30%, respectively (Riefler et al., 2006). Furthermore, it was suggested that the cytokinin-mediated regulation of seed size mostly occurs due to maternal and/or zygotic tissues. Exogenous application of cytokinin has also been proven beneficial in improving the yield-related traits in pulses and oil seed crops. Application of 100–200 µM BAP (6-benzylaminopurine) increased ovule and seed number in *Brassica napus* and also restored the replum development in wild-type *B. napus* and in the *A. thaliana* rpl ntt double mutant (Zuniga-Mayo et al., 2018). Moreover, when different forms of cytokinin *viz.,* [6-benzyladenine (BA), N-(2-chloro-4-pyridyl)-n-phenylurea (CPPU), 6-furfurylaminopurine (KT), and thidiazuron (TDZ)] were applied on *B. juncea*, highest frequency of shoot regeneration was noticed in combinatorial treatment of thidiazuron (TDZ)] and NAA (Guo et al., 2005). Similarly, combination of BAP and IAA resulted in significantly high biomass and seed yield in *Guizotia abyssinica* (L.f.) Cass. (niger seed plant); a multipurpose oil seed crop (Talukdar et al., 2022). Application of 3.4 × 10–7 mol of 6-benzylaminopurine (BA) resulted in a 79% increase in soybean seed yield compared with controls (Nagel et al., 2001).

### Cytokinin-induced/boosting number of seed per pod/pod set/pod development

Among yield-related traits, the number of seeds per/pod and/or number of seed per unit area determines the overall yield (Schou et al., 1978; Kokubun, 1988). The number of seeds per pod or number of seeds produced per unit area is directly proportional to the number of flowers that further develop in to mature pod. Leguminous plants, such as soybean, pigeonpea, and chickpea, produce a higher number of flowers, but most of them abort before reaching maturity (Abernethy et al., 1977). Likely causes include the lack of nutrition, vascular constrictions, and certain hormones (Heindl et al., 1982; Antos and Wiebold, 1984; Brun and Betts, 1984; Kokubun and Honda, 2000). Competition for photosynthesis among seeds and organs is also thought to be a major cause of abortion (Shibles et al., 1975). There are various reports highlighting the exogenous and/or endogenous cytokinin-mediated boost in flower and pod formation (Crosby, 1981; Peterson et al., 1984; Dyer et al., 1987; Mosjidis et al., 1993; Nagel et al., 2001; Yashima et al., 2015).

**FIGURE 2:**
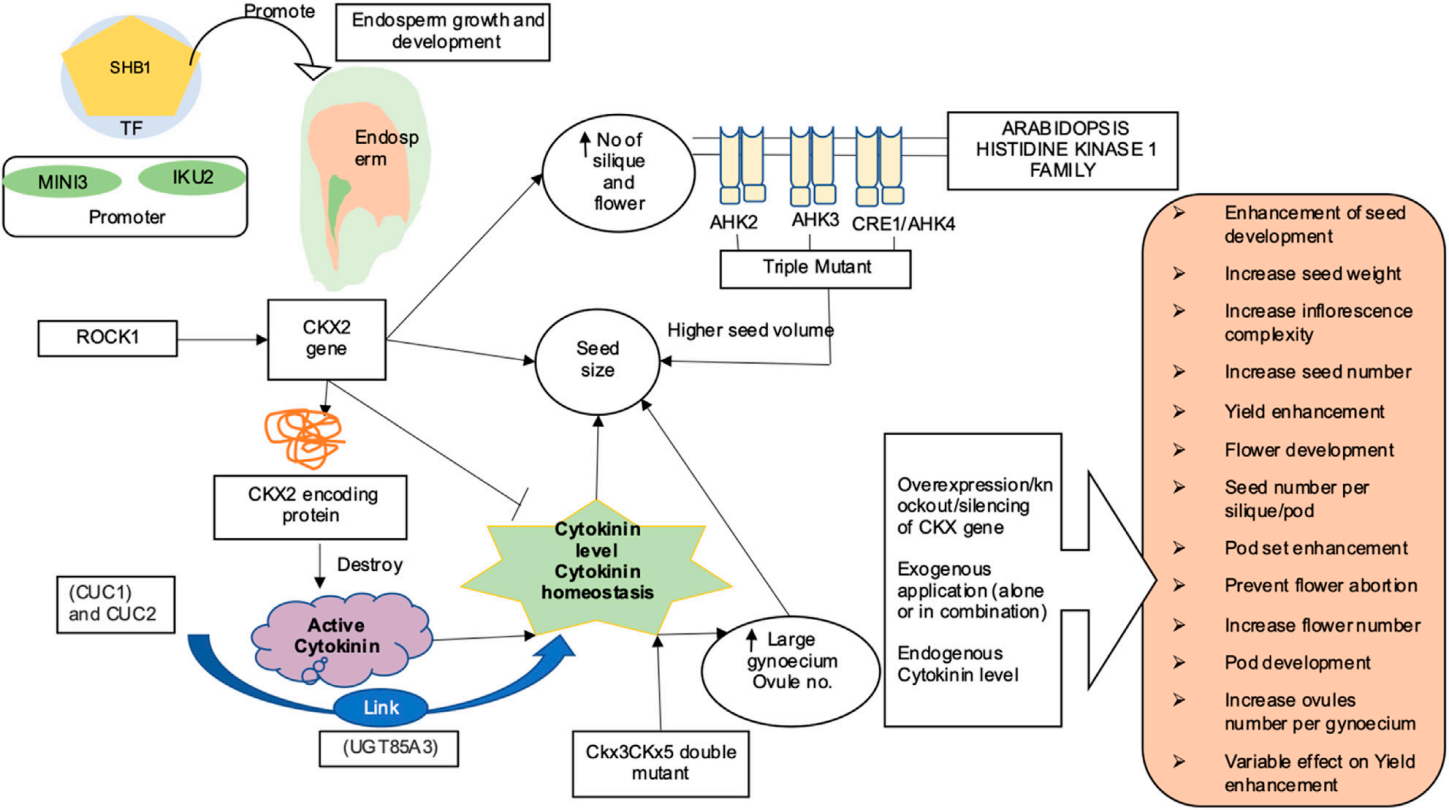
Cytokinin-mediated regulation of yield and yield-related traits (SHB1, Short Hypocotyl Under Blue1; ROCK1, Repressor of cytokinin deficiency 1; AHK1, Arabidopsis histidine kinase1 family; *CKX2*, Cytokinin oxidase/dehydrogenase; *CUC*, Cup-shaped cotyledon; *MINI3*, *MINISEED 3*; *IKU2*, *HAIKU2*) (Riefler et al., 2006; Zhou et al., 2009; Li et al., 2013). Various strategies such as overexpression of gene *CKX/IPT* (Werner et al., 2003), knockdown of *CKX* gene family (Ashikari et al., 2005), gene silencing (Heyl et al., 2008; Schoor et al., 2011), and exogenous application of CK (Nagel et al., 2001; Liu et al., 2018; Talukdar et al., 2022) have been attempted to enhance yield-related trait in pulses and oilseed crop. Dynamic role of cytokinin in orange box-in Arabidopsis; CKs are involved in formation of more number of flower, silique, seed (Bartrina et al., 2011; Niemann et al., 2015), ovule number, and seed yield (Werner et al., 2003; Cucinotta et al., 2018; Nguyen et al., 2021b; Zu et al., 2022) In soybean, CKs are involved in preventing flower abortion and in pod setting (Nonokawa et al., 2012), associated with seed development and fatty acid biosynthesis (Nguyen et al., 2016), increase seed yield (Kambhampati et al., 2017), pod set and seed filling (Jameson and Song 2016; Kambhampati et al., 2017; Cai et al., 2018; Liu et al., 2018), and reduction in floral abscission and increase pod number and seed weight (Carlson et al., 1986; Nagel et al., 2001) In *Brassica* increase in seed number per ovule number, pod number, seed weight (Zuniga-Mayo et al., 2018; Schwarz et al., 2020), more pod number, and increase in seed yield was observed (Atkins et al., 2011)

Figure 2 highlights various aspects of yield and yield-related traits as mediated by cytokinins. Increase in pod number was observed after application of exogenous BA4 to the floral raceme of mung bean (Clifford 1981) and two soybean cultivars (Crosby, 1981; Peterson, et al., 1984) The cultivar Shore, exhibited significant increases in pod number than Essex soybean cultivar. It was speculated that the difference in response was due to Shore ovules having a lower endogenous level of cytokinin-like activity than that in Essex ovules at the time of BA application. Similarly, the administration of three BA on the top of the nodes of field grown soybean resulted in an increase in total number of pod per plant by 27% and seed weight by 18% (Carlson et al., 1986). Furthermore, an increase in the total number of flowers, pods, and number of ovules per gynoecium observed in soybean and oilseed rape after exogenous application of cytokinin (Dyer et al., 1987; Honig et al., 2018) showed that the cytokinin system could be effectively utilized as a target for improving yield and yield-related traits in dicots as well. Bartrina et al., 2011 and Gailbiati et al., 2013 reported that an increased cytokinin levels in the *ckx3 ckx5* double mutant result in a larger gynoecium and production of more ovules. Similarly, sextuple *ckx3 ckx5* mutants were observed to have higher cytokinin concentrations with larger and highly active inflorescence meristems. They also produced up to 72% more flowers on the main stem, with the gynoecia had 32% and 54% more ovules pods, respectively. In addition, the weight of seeds extracted from the main stem of plants was found to be heavier by 20–32% (Schwarz et al., 2020). Furthermore, cytokinins have been shown to increase ovule quantity in other Brassicaceae species, implying that genetic manipulation of cytokinin metabolism could be an effective technique for increasing seed yield (Cucinotta et al., 2016; Zuniga-Mayo et al., 2018) Surprisingly, it was known recently that high amounts of cytokinin hindered fruit elongation in *ckx7* mutants (Di Marzo et al., 2020). Niemann et al., 2015 highlighted the formation of more number of flowers and siliques as a result of mutation in the *ROCK1* gene (*REPRESSOR OF CYTOKININ DEFICIENCY1*), which is essential for full *CKX* function. Furthermore, in the *ugt85a3* (*UDP-GLUCOSYL TRANSFERASE 85A3*) mutant, lower cytokinin inactivation resulted in the development of more ovules per gynoecium (Cucinotta et al., 2018). In soybean, higher levels of cytokinin were found in the reproductive tissues during the pod set and seed filling phases (Jameson and Song, 2016; Liu et al., 2018; Zuniga-Mayo et al., 2018). Metabolite profiling of 27 cultivars of field-grown soybeans (pod and seed tissues) revealed that high producing varieties maintained a constant supply of cytokinins *via de novo* biosynthesis into later stages of development as compared to low yielding soybean genotypes. In addition, zeatin-type cytokinins are required for pod/seed set, whereas isopentenyladenine-type cytokinins have a role in seed filling (Kambhampati et al., 2017). Numerous studies in various crops have reported the significant impact of cytokinins in mediating yield and its related traits through a range of studies, such as exogenous cytokinin application, NGS, transgenic expression studies, advanced chromatographic techniques, mass spectrometry, and many others (Table 1).

**TABLE 1:** Impact of cytokinins on yield and yield-related trait in oilseed and pulses.

Trait under study	Crops	Cytokinin types/Conc	Methodology used/parameter estimated	Results	References
Other traits	*Arabidopsis thaliana*	NA	Cytokinin response assay and *v*arious inhibitors of known signaling pathways were tested	Primary alcohols that specifically inhibit phospholipase D (PLD) partially prevented cytokinin-induced GUS activity and reduced the accumulation of ARR5 gene transcripts	Romanov et al. (2002)
Yield-related traits	*Arabidopsis thaliana*	NA	*AtCKX-*overexpression	Plants with increased number of flowers and siliques, small leaf buds and apical meristems, and expanded root system	Werner et al. (2003)
Other traits	*Arabidopsis thaliana*	NA	Overexpression of an aldose-like enzyme (ALL)	Elevated CK signaling (increased ARR4 and ARR5 expression), dwarfism, reduced apical dominance, and dark green rolled leaves	Jung et al. (2005)
Other traits	*Arabidopsis thaliana*	NA	Gene silencing 35S:ARR1-SDRX	Insensitivity to active CKs arising from loss of the B-type Arabidopsis response regulator 1 *via* gene silencing	Heyl et al. (2008)
	*Arabidopsis thaliana*	NA	Induced mutations in ↓*Atckx3ckx5*	Formation of more no of flower, Silique number and seed number	Bartrina et al. (2011)
Other traits	*Arabidopsis thaliana*	NA	SiRNA- and artificial miRNA-mediated silencing of ADK (adenosine kinase) Comprehensive HPLC-tandem MS analysis	In ADK-deficient roots and leaves, cell division was irregular. The metabolic studies of ADK-deficient lines revealed an irregular organization of root tip and root cap cells, decreased meristem diameters, and expanded cells in the elongation zone, highlighting the importance of ADK in CK homeostasis *in vivo*	Schoor et al. (2011)
Yield-related traits	*Arabidopsis thaliana*	NA	Mutation in the *ROCK1* gene (*REPRESSOR OF CYTOKININ DEFICIENCY1*)	Enhanced SAM activity and formation of more number of flowers and siliques	Niemann et al. (2015)
Yield-related traits	*Arabidopsis thaliana*	NA	Functional characterization of *UDP-GLUCOSYL TRANSFERASE 85A3 (UGT85A3)* and *UGT73C1*	*CUP-SHAPED COTYLEDON1* (CUC1) and CUC2 regulate cytokinin homeostasis by interacting with UGTs to determine ovule number thus seed yield	Cucinotta et al. (2018)
Yield-related traits	*Arabidopsis thaliana*	6-BA and eBL 10 mmol/L (30 min 1 μmol/L eBL and 30 min 1 μmol/L BRZ)	Crossed BR-and CK-related mutants to test if these two phyto-hormones functions together in ovule initiation	Increasing BR and CK levels at the same time resulted in more ovules and seeds than increasing BR or CK individually. *BZR1*, a BR-response transcription factor, interacted directly with ARR1, to increase ovule initiation. Brassinosteroid-cytokinin interaction improved ovule initiation and increases seed quantity per silique	Zu et al. (2022)
Other traits	*Arabidopsis thaliana*	NA	*CYTOKININ-RESPONSIVE GROWTH REGULATOR (CKG)*, mediates CK-dependent regulation of cell expansion and cell cycle progression in *Arabidopsis thaliana*	From embryonic through reproductive phases, CKG promoted organ development in a pleiotropic manner, especially in cotyledons. Conversely, cotyledons were smaller in ckg loss-of-function mutants. CKG primarily controls the expression of cell cycle-related genes such as *WEE1* (a cell cycle promoting factor)	Park et al. (2021)
Yield-related traits	*Arabidopsis thaliana*	NA	Impact of an altered epidermal cytokinin metabolism on Arabidopsis shoot development	This cytokinin action was primarily mediated by the AHK3 receptor and the transcription factor ARR1.Increased cytokinin production in the outer layer of reproductive tissues and the placenta resulted in the placenta producing more ovules and longer siliques. As a result, more seeds in longer pods, leading to higher seed yield per plant	Werner et al. (2021)
Other trait	*Arabidopsis thaliana*	NA	The effect of light intensity on the cold response in *Arabidopsis thaliana*	Transcription of genes related to CK metabolism and signaling showed a tendency to re-establish, CK homeostasis in both transformants. Up-regulation of strigolactone-related genes indicated their role in suppressing shoot growth. The analysis of leaf proteome revealed over 20,000 peptides, representing 3,800 proteins and 2,212 protein families	Prerostova et al. (2021)
Oilseed and pulses					
Yield-related traits	Oilseed Rape	NA	Measurement of various cytokinin during pod development with high performance liquid chromatography and immunoenzymic (enzyme-linked immunosorbent assay, ELISA) techniques	Variable effect on yield enhancement were noticed such as increase flower number, increase ovules number per gynoecium and pod development	de Bouille et al. (1989)
Yield-related traits	Oilseed Rape (*Brassica napus L*.)	NA	Constitutive expression of *IPT* gene under Slightly leaky maize heat-shock (hsp70)	Increase in seed number and seed weight were found	Roeckel et al. (1997)
Shoot regeneration	Oilseed Rape (*Brassica juncea* var.)	[6-Benzyladenine (BA), *N*-(2-chloro-4-pyridyl)-*n*-phenylurea (CPPU), 6-furfurylaminopurine (KT) and thidiazuron (TDZ)]	The shoot regeneration frequency of cotyledon and leaf	The highest frequency of shoot regeneration was 61.3%–67.9% in cotyledon and 40.7%–52.4% in leaf segment respectively when 2.27 or 4.54 μM TDZ was combined with 5.37 μM NAA	Guo et al. (2005)
Leaf senescence and yield	Canola (*Brassica napus L.*)	NA	Regulated Expression of a IPT gene using AtMYB32 promoter Evaluation of seed quality parameters; fatty acids (% of oil content)	The yield was increased from 16 to 23%. Oleicacid content was increased in all transgenic lines, with higher oil content and reduced glucosinolate levels in one particular transgenic line. Increase the number of flowers, siliques, and overall yield	Kant et al. (2015)
Pod development and stress responses	Oilseed Rape (*Brassica napus L.*)	Cytokinin 6-benzylaminopurine (6-BA) and the auxin indole-3-acetic acid (IAA)	Genome-wide identification and expression profiling of *CKX* Genes	A total of 23 BnCKX genes were identified and the expression levels of *BnCKX5*-1, 5–2, 6–*1*, and 7–1 significantly differed between the two lines and changed during pod development. Also exhibited role in increasing silique length and pod development	Liu et al., (2018)
Flower and fruit development traits	*Brassica napus and Arabidopsis thaliana*	100–200 µM BAP (6-benzylaminopurine	Hormone treatment, microscopy, parameters related with fruit development	Cytokinin affects stamen filament elongation and anther maturation, and causes a conspicuous overgrowth of tissue in petals and gynoecia. Also increases in ovule and seed number was observed	Zuniga-Mayo et al. (2018)
Yield-related traits	*(Brassica napus)* Mutant *ckx3* and *ckx5*	NA	RNA-seq analysis and *in situ* hybridization	Increased cytokinin concentration and larger inflorescence meristems. Increase in no of flowers, ovules, no of pods and seed weight were noted	Schwarz et al. (2020)
Yield-related traits	Chickpea (*Cicer arietinum*)	NA	Estimation of cytokinin at four developmental stages in chickpea using gas chromatography–mass spectrometry	Enhancement of seed development and Increase seed weight	Emery et al. (1998)
Yield-related traits	*Guizotiaabyssinica* (L.f.) Cass. (Multipurpose oil seed crop)	6-Benzyl aminopurine (BAP) 25, 50, 75, and 100 mg L^−1^	Physio-chemical properties of soil of experimental site, FA composition and yield-related traits in (niger seed plant)	The combination of IAA (50 mg L^−1^) and BAP (100 mg L^−1^; I_50_B_100_) yielded significantly high biomass (38 and 40 g plant^−1^) and seed yield (13.24 and 12.67 g plant^−1^) in 2014 and 2015, respectively	Talukdar et al. (2022)
Yield-related traits	Lupin	NA	Flower-specific expression of *IPT* gene	More pod number and increase in seed yield was observed	Atkins et al. (2011)
Yield-related trait	Soybean (*Glycine max*)	Applications of BA	Growth characteristics and agronomic traits, including abscission, pod number and seed weight	Reduction in floral abscission and increase in total pod number and seed weight by 27 and 18%, respectively	Carlson et al. (1986)
Yield flower and pod Set	Soybean (*Glycine max*)	6-benzylaminopurine (BA)	Number of pods, seeds per pod, and the total seed weight per plant were measured	In the greenhouse, application of 3.4 × 10^–7^ mol of BA resulted in a 79% increase in seed yield compared with controls. Pod set enhancement and increase seed weight	Nagel et al. (2001)
Yield-related trait pod and seed development	Soybean (*Glycine max*)	2-(2,4-dichlorophenoxy) propanoic acid (2,4-DP) and 6-benzylaminopurine (BAP),0.12mM, 0.08 mM, 0.04 mM, and 1.5mM, 1 mM, 0.5 mM	Determination of patterns of flower, pod and seed development. Association of reproductive abscission with growth characteristics, including seed yield and weight in two genotypes	BAP (0.5 mM) dramatically decreased flower abortion and delayed pod abscission, leading in higher pod setting rates. 1 mM BAP raised 100-seed weight to 22.3 g at R1 in Manlee (big seeded) and 11.9 g at R3 in Pungsan under field circumstances utilizing intermediate concentrations. BAP (1 mM) at R3 in Pungsan (small seeded) considerably boosted seed yield (40.1 g plant1)	Cho et al. (2002)
Yield-related traits	Soybean (*Glycine max*)	Cytokinin (6-benzylaminopurine, BA)	The endogenous cytokinin (transzeatin riboside) content of individual florets was measured at the 1, 3, 5, 7th position every 3 days after anthesis and the pod-set%age were calculated in racemes of soybean genotype IX93-100	Cytokinin was detected only from the florets at 9 DAA, and the content was higher in the more proximal florets than in the 7th floret. These findings imply that increasing the quantity of cytokinin in individual florets may improve the pod setting of the florets positioned at the middle or distal part within the raceme	Nonokawa et al. (2012)
Yield	Soybean (*Glycine max*)	NA	Identification and quantification CK using (HPLC–MS/MS) at three stages of reproductive development in 27 cultivars of Glycine max	Levels of cytokinins strongly correlated with yield and associated traits at stages critical for reproductive development. Isopentenyladenine type cytokinins increase seed filling whereas zeatin type cytokinins exhibited role in pod/seed set.	Kambhampati et al., (2017)
Yield and biological nitrogen fixation	Soybean (*Glycine max*)	Cytokinin was applied (seed or foliar	Nitrogen source, use efficiency and harvest index, tested in two commercial soybean genotypes (DM50I17 and DM40R16)	In the field, DMR50I7 achieved consistent yields across sowing dates because increased Biological Nitrogen Fixation compensated for limited soil N uptake in early sowing dates, also leading to 25% higher nitrogen use efficiency (NUE)	Kempster et al. (2021)
Cytokinin content and seed yield	Soybean (*Glycine max*)	NA	Genome-wide identification and expression profiling of *CKX* Genes and CK metabolite profiling	A total of 7 *GmCKX* GFMs were identified. Natural variations in SNP were found in five of the seventeen identified *GmCKX GFMs.* Soybean lines with this mutation exhibited higher CK content and desired yield characteristics	Nguyen et al., (2021a)
Other traits	*Medicago truncatula*	Cytokinin 6-benzylaminopurine (6-BA) and indole-3-acetic acid (IAA)	Genome-wide identification and expression profiling of *CKX* Genes	A total of 9 putative *CKX* homologues were discovered. Disruption of Medtr4g126160, which is mostly expressed in roots, resulted in reduction in primary root length and increase in lateral root number, showing the specific roles of cytokinin in regulating root architecture	Wang et al. (2021)
Yield-related trait	Soybean (*Glycine max*) and Cowpea (*Vigna unguiculata*)	NA	Integrated Bioinformatics Analyses of *PIN1*, *CKX*, and yield-related genes for the trait seed number per pod	Although the two genes involved in embryo development interact with the *CKX* gene family, VuACX4 demonstrated a substantially higher relative expression level than *GmACX4.* Following then, a tandem duplication in legumes resulted in the separation of *CKX3* into *CKX*3a and *CKX3b*, with *CKX3a* being a critical gene controlling ovule number	Liu et al. (2021)

**FIGURE 3:**
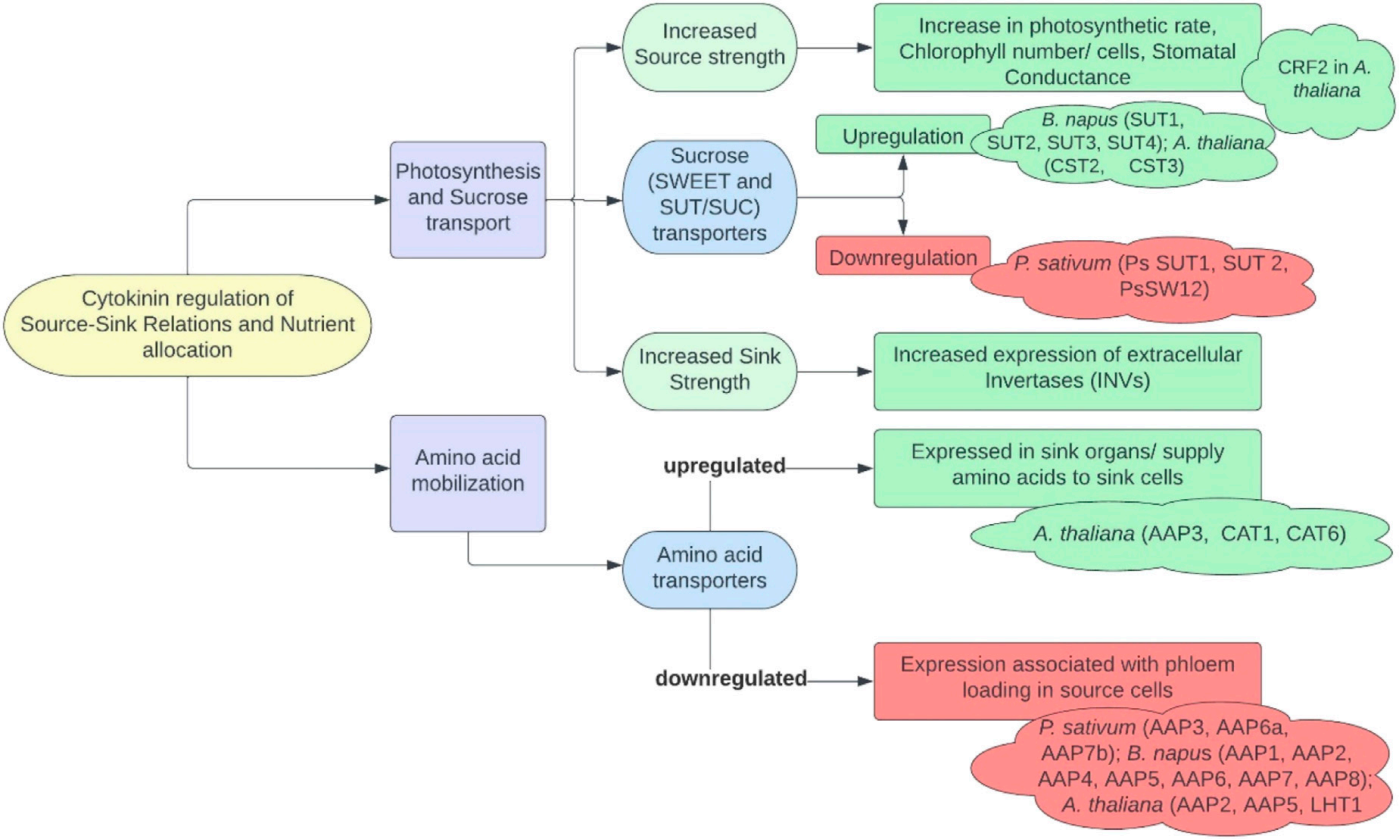
Insights into cytokinin-mediated source-sink relationship and nutrient allocation (SWEET, SUGARS WILL EVENTUALLY be EXPORTED TRANSPORTERS; SUT/SUCs, SUCROSE TRANSPORTERS/CARRIERS; *AAP*, amino acid permease; *LHT*, lysine and histidine transporters; *CAT*, cationic amino acid transporters) (for references, see McIntyre et al., 2021; Harms et al., 1994; Ninan et al., 2017; Song et al., 2015; Ehness and Roitsch, 1997; Godt and Roitsch, 1997; Yang et al., 2014; Chernyad’ev, 2000; Ahanger et al., 2020; Boasson and Laetsch, 1969; Okazaki et al., 2009; Lee et al., 2007; Kiba et al., 2011; Kiba et al., 2005; Yokoyama et al., 2007; and Jian et al., 2016)

Source-sink pathways are always in as dynamic state during the entire life cycle of a plant. Initially starting their life cycle as sinks, leaves mature into source for the seeds, with the latter acted upon as a source to begin with, that is, providing energy and nutrients during germination. Central to these relationships are the availability and partitioning of two major resources: carbon and nitrogen. Sucrose and amino acid mobilization are controlled by *SWEET* (sugars will eventually be exported) and *SUT* (sucrose transporters)/*SUCs* (sucrose carriers) transporters and cell wall invertases for the former and primarily *AAPs* (amino acid permease) for the latter. Cytokinins have also been observed to regulate sink number as well as sink size in various legumes, cereals, and Arabidopsis. These signaling molecules are reported to increase the strength of sink tissues and magnetize the assimilates through either influencing sucrose metabolism and transport or promoting cell division (Brenner and Chiekh 1995; Emery and Atkins 2006) through upregulation of cyclins controlling check points of cell cycle (Riou-Khanlichi et al., 1999) and increased phloem unloading in seed coat (Brenner and Chiekh 1995). The insight into cytokinin-mediated influence on source-sink relations and nutrient allocation is depicted in Figure 3. Developing seeds have been documented with the highest cytokinins level among all plant tissues; thus, they are rich source of the former metabolites (Emery et al., 2000). As reported in *Arabidopsis*, *IPT* gene expression in the endosperm continues till early heart stage of its developing seeds, making them sites of cytokinin biosynthesis (Miyawaki et al., 2004), with minor quantities being translocated from xylem or phloem (Emery et al., 2000). Jameson et al., 2016 conducted expression profiling *of SWEET*, *SUT*, *AAP*, *CWINV* (cell wall invertases), *IPT*, *LOG* (Lonely Guy/cytokinin phosphoribosyl hydrolases) and *CKX* genes to ascertain source-sink dynamics in germinating seeds of *P. sativum.* They reported an active expression of cytokinins in imbibing seeds and its biosynthesis in germinating seedlings as well as strong expression of specific genes regulating source-sink dynamics in plants.

Ectopic expression of an IPT gene (isopentenyltransferase) has been observed to increase seed yield. In addition, the inherent expression of cytokinins is highly dynamic and changes rapidly over time, as observed in developing cereal grains (Morris et al., 1993). In wheat, expression profile of specific members of the cytokinin biosynthesis (IPT), degradation (CKX), O-glucosylation, and ß-glucosidase gene families have been implicated in the changing cytokinins levels (Song et al., 2012). In legumes, the level of cytokinins also influence the pod set. Application of cytokinins in lupin has been observed to prevent abortion (Aitkins and Pigeaire, 1993). Emery et al. (2000) investigated correlation of cytokinins with abortion in developing flowers and pod set by increasing an *IPT* gene expression (Atkins et al., 2011). A detailed GC-MS analysis on developing white lupin revealed the expression nature of cytokinins and showed that the peak and transient cytokinin expression occurs in the liquid endosperm of developing seeds (Emery et al., 2000). Several evidences have pointed out the fact that in legumes maternally derived cytokinins are restricted to the pod and seed set (Emery et al., 2000), but in the developing embryo cytokinin biosynthesis is active in the filial tissues (Singh et al., 1988; Emery et al., 2000). In Arabidopsis, Day et al. (2008) observed that the cell cycle genes and cytokinin biosynthesis (IPT8) genes play a crucial role in the syncytial endosperm development.

### Spatial expression of cytokinin gene regulating seed/seed number per pod development

Active sites for cytokinin biosynthesis include developing seeds, pod walls and seed coats. Cytokinins have been demonstrated to influence yield by inducing flowering, increasing silique/seed number/pod, and seed size. Various members of the cytokinin gene family viz cytokinin biosynthesis related gene family (*BnIPT1*, 2, 3, 5, 7, 8, and 9), cytokinin degradation gene family (*BnCKX1* to *BnCKX7*), cell wall invertase gene family (*BnCWINV1* to *BnCWINV6*), sugar transporter gene family (*BnSUT1* to *BnSUT6*), and amino acid permease–related gene family (*BnAAP1* to *BnAAP8*) have been identified in *Brassica napus* as a target for breeding (Song et al., 2015; Ninan et al., 2017). It has been reported that developing seeds are the major site of cytokinins and the filial tissues of developing legume seeds have been shown to rely on cytokinin biosynthesis (Singh, 1988; Jameson et al., 2016), whereas pod walls and seed coats have been observed to have significant amounts as well as different forms of cytokinin (Davey and van Staden, 1977, 1978, 1979; Zhang and Letham 1990; Emery et al., 2000; Song et al., 2015). In *Arabidopsis*, many studies have observed dynamic and differential spatiotemporal expression patterns of *IPT* gene family members (Miyawaki et al., 2004; Belmonte et al., 2013) as well as in other members of Brassicaceae family (O’Keefe et al., 2011; Liu et al., 2013). After profiling all seven *AtIPT* gene family members using RT-PCR, Miyawaki et al. (2004) observed the presence of the cytokinin biosynthesis pathway in most plant organs. However, distinct tissue specificity was observed in reporter gene (GUS) constructs used. But still, each family member was found to be expressed at several regions. Liu Z. et al. (2013) observed *BrIPT1*, *3*, *5*, and *7* genes to be strongly expressed in the roots, whereas *BrIPT8-1* was largely confined to immature siliques and *BrIPT8-2* in stamens of *Brassica rapa*. These evidences match well with the data on the developing seeds of the *Arabidopsis*, where expression of genes like *AtIPT8* and *AtIPT4* is localized to the chalazal region (Miyawaki et al., 2004; Day et al., 2008; Belmonte et al., 2013).

### Cytokinin and seed germination.

Crop stand and productivity is a manifestation of seed germination and seedling establishment, with the germination process categorized into three phases (water uptake by seeds; mobilization of food reserves and reactivation of metabolism; and radical protrusion) and involves numerous physiological, morphological, and biochemical changes upon favorable conditions, which are regulated by endogenous and exogenous factors. One of the important internal components affecting germination is hormones. Emergence of seeds and buds from dormancy involves reduction in the levels of inhibitors and gradual buildup of growth promoters; thus, extensive changes in seed metabolome repertoire. Role of hormones in mediating a shift of an inert quiescent embryo to a rapidly metabolizing system, that is, seed germination and post germinative seedling growth is well elucidated, particularly that of abscisic acid (ABA) and gibberellins. However, several studies have implicated the regulatory role of cytokinins in seed germination. Different forms and activity of cytokinin are a function of developmental stages, tissues, and plant species in question (Kieber and Eric Schaller, 2010). Cytokinin application was observed to revert the ABA-induced inhibition of seed germination in *Brassica oleraceae* (Khan, 1971), suggesting permissive role of these hormones in removing the blocks (inhibitors) present in seeds. However, the study also suggested that if these inhibitors are absent, role of cytokinins to mediate germination becomes redundant. Water imbibition by seeds exclusively determines radical emergence; however, seedling growth is a feature of remobilization of stored food reserves to zones of growth and mitosis. Several research studies have implicated both of the latter processes to be possibly regulated by cytokinins (Gepstein and Ilan, 1980; Hepler, 1986; MacGaw and Borch, 1995). For instance, reserve material mobilization in *Cicer arietinum* seeds coincides with the period of supply of cytokinin from the embryonic axis to the cotyledons, that is, first 12 h s after the start of imbibition (Martin et al., 1987; Pino et al., 1991). Exogenous cytokinin application has been known to influence the development of embryonic axis as cited by several researchers (Tzou et al., 1973; Alvarez et al., 1987). Gallego et al., 1991 revealed variation in the endogenous level and compartmentalization of different cytokinin groups in embryonic axis of *C. arientinum* L. in response to exogenous cytokinin application and calcium treatment during germination. Zeatin, 2-isopentyl adenosine, and 2-isopentyl adenine application induces germinative changes peculiar to those under normal conditions, such as delay in epicotyl emergence and short and thick embryonic axis with reduced dry weight, whereas application of zeatin riboside and dihydro derivatives did not induce such changes. Furthermore, controlled conditions revealed high amounts of conjugated cytokinins (storage and inactive forms) in the basal regions of epicotyl and hypocotyl of embryonic axis, which are hydrolyzed to free bases followed by their transportation to apical zones, wherein their transformation to dihydro derivatives (most stable form) takes place, as the later form is resistant to the action of cytokinin oxidase enzyme present in the embryonic axis (MacGaw and Burch, 1995). However, a germination medium supplemented with calcium increased the level of dihydro derivatives, whereas exogenous cytokinin application leads to faster appearance but lower levels of these derivatives in embryonic axis segments displaying maximum growth. The later could be attributed to the fact that exogenous cytokinin could lead to stress or produce compounds resistant to enzymes (Whitty and Hall 1974).

**FIGURE 4:**
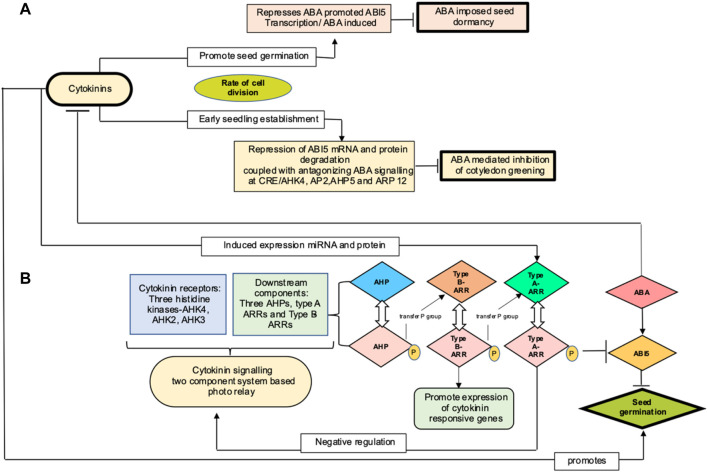
**(A)** Proposed mechanism for cytokinin action in regulating seed germination in *Arabidopsis* by Wang et al. (2011) and Guan et al. (2014), represented at top (light pink (marginal mechanism) and yellow (major mechanism) color boxes, respectively). Cytokinin influences cell division to promote seed germination and seedling establishment and thereby activates ABA-imposed seed dormancy and inhibition of cotyledon greening. **(B)** represents cytokinin signaling components and their interaction with *ABI5* to mediate seed germination (AHK, *Arabidopsis* histidine kinases, ARRs, *Arabidopsis* response regulators; ABA, abscisic acid; *ABI5*, abscisic acid insensitive protein 5)

*ABI5* (abscisic acid insensitive 5) encodes for a basic leucine zipper transcription factor that is best characterized as a key component involved in ABA signaling and early seedling development. ABI5 transcripts buildup during germination and degrade to basal amount post seed germination (Lopez-Molina et al., 2001; Cho et al., 2002). A total of two mechanisms have been proposed wherein cytokinin targets ABI5 to regulate seed germination, as shown in the Figure 4. One route explains marginal regulation by cytokinin through repression of ABI5 expression in an ABA-independent manner (Wang et al., 2011). Second mechanism pertains to the role of cytokinin in promoting plastid differentiation. In *Arabidopsis*, Guan et al. (2014) demonstrated that cytokinin mediates ABI5 protein degradation, thereby alleviating ABA-induced inhibition of cotyledon greening, a focal point during post germination growth and marking the transition from heterotrophy to autotrophy and establishing photosynthetic capacity. In cytokinin signaling, hybrid histidine protein kinases (AHKs) autophosphorylate upon sensing cytokinins through receptor binding. This phosphorelay system comprises of transfer of the phosphate group from the receptors to the downstream components, initially to AHPs (Arabidopsis histidine phosphotransfer proteins), followed by phosphorylation and activation of Myb transcription factor encoding type B ARRs (response regulators). The latter induces expression of cytokinin response gene, including type A ARRs as characterized in *Arabidopsis* (Nishimura et al., 2004; Riefler et al., 2006). When phosphorylated, type A ARRs, through unknown mechanism, negatively regulates cytokinin signaling, thus forming a feedback regulatory loop (Muller and Sheen 2007; To and Kieber 2008; Hwang et al., 2012). To et al. (2007) and Ren et al. (2009) reported cytokinin to positively regulate steady level of type A- ARRs mRNA by increasing the stability of protein in a phosphorylation-dependent manner and further postulated type A ARR–ABI5 complex to inhibit *ABI5* protein interaction with proteasome degradation. Cytokinin promotes seed germination by mimicking the action of auxin, stimulating *ABI5* protein degradation and thus uplifting the abscisic acid inhibitory effect on post germination growth of *Arabidopsis*. Thereby, degradation of *ABI5* protein rather than its mRNA is a major step in cytokinin-mediated ABA signaling.

Convergence of cytokinin with light signaling has been demonstrated through interaction of type-A ARRs with phytochrome b (phyb) (Sweere et al., 2001) and a bZIP transcription factor, HY5, in *Arabidopsis* (Ang et al., 1998). Hutchison et al. (2006) and Riefler et al. (2006) emphasized role of cytokinin receptor genes and *AHPs* in far red light regulated seed germination, implicating cytokinin repressed ABA signaling as an important regulatory mechanism to coordinate early seedling establishment. An enhanced cell division rate as a function of seed priming with cytokinin, particularly kinetin has also been related to improved germination and robustness of seeds (Sawan et al., 2000; Tahaei et al., 2016). Wang et al., 2011 characterized *Arabidopsis gim1* (germination insensitive to ABA mutant 1) mutants’ deficit for *AtIPT8* gene encoding for isopentenyl transferases, catalyzing a rate limiting step in the cytokinin biosynthetic pathway (Sun et al., 2003; Miyawaki et al., 2006). These mutants were characterized for reduced expression of *ABI5* gene, and the expression could not be restored with exogenous ABA treatment. However, ectopic expression of *AtIPT8* (ecotypic expression) in OE-2/Com1 transgenic plants was observed to raise the cytokinin level and ABA insensitive seed germination features of *gim1* mutants was observed.

Several advanced studies have also been conducted to elucidate the role and establish mechanism of cytokinin in germination of oil seed crops and legumes. For instance, first study underpinning legume metabolism in relation to seed germination was conducted by Araujo et al. (2019). Priming of *Medicago truncatula* seeds (50 mM) kinetin throughout germination followed by detailed metabolome and physiological characterization was observed to speed up radical protrusion caused an impairment of seedling growth at the root level. Kinetin affected content of 27 metabolites at radicle emergence stage, chiefly associated with rapid decline of metabolites linked to germination and stress indicating the role of kinetin as both stress agent and inducer of seed germination. Such targeted studies hold potential in identifying the point, wherein priming of cytokinin needs to be stopped for preventing genotoxicity. However, Riefler et al. (2006) demonstrated role of cytokinin in seed germination, shoot and root development, seed size, and senescence through loss of function mutants for cytokinin receptors (*AHK2*, *AHK3*, and *CRE1/AHK4*) in *Arabidopsis*. Rapid germination, decreased sensitivity to far-red light and increased dark germination were observed in the mutants, revealing functions of these cytokinin receptors in regulating these processes. Cytokinin, thus, was observed to negatively regulate light-dependent seed germination in *Arabidopsis*. Similarly, Oh et al. (2009) conducted genome wide chromatin immunoprecipitation (ChIP)—chip analysis in *Arabidopsis* targeting role of phytochrome interacting factor 3–*LIKE5* (*PIL5*), a basic helix-loop-helix transcription factor (TF) in seed germination. It was observed that phytochromes when activated mediated degradation of *PIL5* and enabled seed germination. *PIL5* was identified to directly or indirectly regulate gene expression of several hormonal signaling networks, including GA, ABA, JA, ethylene, and cytokinins, and also impacting the expressions of various genes encoding cell enzymes involved in cell wall modification. Of interest, PIL 5 was observed to directly downregulate expression of cytokinin response factors, that is, *CRF1*, *CRF2*, and *CRF3* genes as well as to upregulate the expression of *AHP5* gene. *CRFs* (AP2 domain TFs) and *AHP5* are known to positively upregulate subset of cytokinin responses (Rashotte et al., 2006) and cytokinin signaling, respectively; therefore, *PIL5*-mediated reverse regulation of these cytokinin positive signaling genes.

### Future perspectives

It has been observed that when cytokinin levels rise, their degradation also kicks in. This is the one of the most important considerations for researchers aiming to target increased cytokinin content as plants will respond to it by accelerating the cytokinin degradation as a result of active homeostasis. Key participants to cytokinin degradation are the *CKX* catalyzing degradation of several cytokinin forms (Werner et al., 2006; Gajdošová et al., 2011). Table 2 shows the expression of the members of *IPT* and *CKX* gene family in different tissues of oilseed and pulses. These gene families have been the preferred targets for yield improvement in several crops. It has been shown that natural or artificial induction of cytokinins result in increased activity of *CKX* (Brugière et al., 2008; Motyka et al., 2003; Liu et al., 2013a, Liu et al., 2013b). Pertinently, a detailed characterization of the spatiotemporal expression of cytokinin gene family need to be performed in oilseeds and pulse crops for shortlisting significant genes that can be further targeted for genome engineering with the aim to boost the yield. However, appropriate genome editing strategies need to be developed as genes involved in cytokinin biosynthesis and its catabolism belong to multigene family and are pleiotropic in nature.

**TABLE 2:** Tissue-specific expression of genes involve in seed/silique per pod/seed development.

Gene name	Crop	Tissue (site of expression)	References
BrIPT1, 3, 5, and 7	Field mustard (*Brassica rapa*)	Expressed in root	Liu et al. (2013b)
BrIPT8_1		Expressed in immature siliques	
BrIPT8-2		Expressed in stamens	
BnIPT1-2/1–3 and BnIPT8-1/8–3		Expressed in siliques and developing seeds	
tRNA IPT genes, BrIPT2, BrIPT9-1, and BrIPT9-2		Ubiquitously expressed	
BrIPT1-1 and BrIPT1-2		High expression in small and medium-sized buds while low expression in big bud	
BrIPT7-1 and BrIPT7-2		Expressed in stamen and root	
BrIPT5-1 and BrIPT5-2		Expressed in root	
BrIPT8-1		Highest expression in siliques	
BrIPT8-2		Mainly expressed in stamens	
*BrCKX7-1* and *BrCKX7-2*		Uniformly expressed at high levels in sepals and petals	
*BrCKX1-1*		Expressed in root	
*BrCKX1-2*		Expressed in stamen, flower, and petal	
*BrCKX3- *		Highly expressed in petals, stamens, and flowers	
*BrCKX3-2*		Mainly expressed in the floral buds	
*BrCKX4*		Highly expressed in root	
BrCKX5		Highly expressed in stamen	
BrCKX6		Expressed in root, leave, and sepals	
BnIPT1		Not detected in immature siliques	
BnIPT8		Expressed in developing siliques and low expression in seed maturation	
BrCKX2-2		Expressed in reproductive tissues	
BnCKX2-1 and 2–2		Expression was restricted to siliques and seeds	
*BnCKX5-1* and *5–2*	Oilseed brassica (*Brassica napus L.*)	Expressed in seeds and silique pericarps	Liu et al. (2018)
*BnCKX6-1* and *6–2*		Expressed in leaves, stems, and silique pericarps but were not expressed in seeds or buds	
*BnCKX7-1*		Highly expressed in stems and leaves	
BnCKX3-1 and 3–4		Expressed specifically in buds and seeds	
AtIPT8 and AtIPT4	*Arabidopsis thaliana*	Expressed in chalazal region of developing seeds	Miyawaki et al. (2004), Day et al., (2008), Belmonte et al. (2013), Kakimoto, (2001), Liu et al. (2013b), Werner et al. (2003), Werner et al. (2006); Mason et al. (2005); Yokoyama et al. (2007); Tajima et al. (2004), Miyawaki et al. (2004), Ye et al. (2002); Takei et al. (2004), D’Agostino et al. (2000), Kiba et al. (2002), Ferreira and Kieber. (2005), Yokoyama et al., (2007)
AtIPT1		Expressed in siliques, integument and seed coat of immature seeds	
AtIPT6		Regarded as pseudogenes	
AtCKX1		Expressed at lateral root junction	
AtCKX1 and 5		Expressed in young floral tissue	
AtCKX1, 2, and 4		Expressed in trichome	
AtCKX6		Expressed in leaf vasculature and root vasculature	
AtCKX4		Expressed in root cap	
AtCKX4 and 6		Expressed in stomata	
AtCKX5		Expressed in root procambium and axillary bud	
AtCKX5 and 6		Expressed in root primordium and mature floral tissue	
ARR5, 8, and 9		Expressed in root meristem	
ARR1, 2, 10, 11, 12, 18, and 20; ARR5 and 6		Expressed in shoot meristem	
ARR1, 2, 10, 11, 12, 13, 14, and 18		Expressed in young leaf	
ARR 2, 10, and 12		Expressed in lateral root junction	
ARR3, 4, 6, 8, and 9; ARR1, 2, 10, 12, 13, and 20		Expressed in leaf vasculature	
ARR1, 13, 18, and 20		Expressed in mature floral tissue	
ARR20 and ARR21		Expressed in reproductive organs	
ARR1, 13, 18		Expressed in young floral tissue	
ARR5, 8, and 9		Expressed in root cap	
*AtIPT4* and *AtIPT8*		Expressed in developing seeds, highest expression in the CZE	
*AtIPT1*		Expressed in distal part of cotyledons and cell files in the procambium linking to the xylem, root procambium	
*AtIPT1* and *7*		Expressed in mature floral tissue	
*AtIPT5*		Expressed in root cap in primary or lateral roots at their early developmental stages and in root primordia	
*AtIPT1*, *AtIPT5*, and *AtIPT1*		Expressed in lateral buds (areal portion) and axillary bud	
*AtIPT3*		Expressed in phloem	
*AtIPT7*		Endodermis, Trichome	
*AtIPT2* and *AtIPT9*		Ubiquitously with higher expression levels in proliferating tissues	
*VuCKX5*	Cowpea (*Vigna unguiculata*)	Expressed in flowers, roots, and pods	Liu., et al. (2021), Nguyen et al. (2020), Galbiati. et al., (2013), Mens et al. (2018), Nguyen et al. (2021a)
*VuCKX6* and *VuCKX7*		Expressed in root	
*VuCKX3a*		Expressed in flowers	
*PvCKX7-1* and *PvCKX6- *	Kidney bean (*Phaseolus vulgaris*)	Expressed in roots	
PvCKX3a		Expressed in flowers	
GmCKX14	Soybean (*Glycine max*)	Highest expression in all seed developmental stages	
*GmCKX3a*, *GmCKX7-2*, and *GmCKX6-1*		Expressed mainly in flowers	
*GmCKX3b-2* and *GmCKX3b-3*		Expressed in roots	
GmCKX08		Expressed in pod	
GmCKX13		Expressed in vegetative tissue	
GmIPT1 and GmIPT2		Root	
*GmCKX7-1* and *GmCKX1-2*		Expression level in all three seed stages. transcripts of GmCKX7-1 and	
*Ps CKX2*	Pea (*Pisum sativum*)	Highest expression in the pod wall and whole seed (Early stages)	Grant et al. (2021)
*PsCKX 1*		Expressed in 5-day pod wall and 10-day pod wall	
*PsCKX 7*		Highest expression in whole pod including seed 1DAP and also in 7-day and 10-day pod wall	
*Ps IPT1*, *Ps IPT2*, and *Ps IPT4*		Expressed in whole pod including seed 1DAP	
*PsIPT 4*		Expressed in seed coat during developmental stages 12–18 days	
*PsCKX1*, *PsCKX2*, *PsCKX5*, and *PsCKX7*		Throughout seed coat development 12–30 days	
*PsCKX1* and *PsCKX7*		Highly expressed in 30-d cotyledon	

In the past decade, several reviews have focussed on summarizing the role of cytokinins in biotic and abiotic stresses (Cortleven et al., 2019), nitrogen nutrition (Gu et al., 2018), senescence (Zwack and Rashotte, 2013; Honig et al., 2018), and seed yield (Jameson et al., 2016). In addition, the significance of cytokinin dehydrogenase: a genetic target for yield improvement in wheat crop (Chen et al., 2020) has also been extensively discussed. However, the role of cytokinins in oilseeds and pulses has not been reviewed so far. In this review, we have tried to gather all the relevant information concerning cytokinin-regulated yield traits in these crops. Nevertheless, information on cytokinin metabolomics also needs to be reviewed which might open new avenues for cytokinin-targeted research for improving crop yield and quality.

Quantification and qualitative analysis of cytokinins is pivotal to determine their association with agronomically important traits. Several studies have substantiated the relation between yield, associated parameters, and cytokinins and described them as evolutionary conserved and of utmost functional significance. However, advanced multidisciplinary approaches targeting cytokinin purification, profiling, quantification, and underlying molecular mechanisms are required to be undertaken in pulses and legumes, specifically. Omics tools and techniques provides a peek through in cytokinin gene families, spaciotemporal gene expression patterns, and metabolite characterization, to further dissect cytokinin-mediated source-sink relations and associated metabolic pathways in details. Identifying and targeting candidate genes will further pave the road to enhance the endogenous cytokinins level using transgenic and breeding approaches. Cytokinin homeostasis and its regulatory networking could also be fine-tuned in pulses and oilseeds through detailed characterization of its gene families-, tissue-, and stage-specific expression data, detecting novel mutations and their applicability to harness the potential of techniques such as MAS, TILING, and CRISPR and many more for developing new germplasm, breeding lines, and varieties with maintained quality traits. Comprehensive depiction of the inhibitors and activators of key genes of cytokinins homeostasis, such as *CKX* and *IPT*, and standardization of studies conducting their exogenous applications could also be utilized for enhancing abiotic stress tolerance as well as *in vitro* organogenesis and holds immense potential toward micropropagation in numerous agricultural, horticultural crops, and in forestry.

## Data Availability

The original contributions presented in the study are included in the article/Supplementary Material; further inquiries can be directed to the corresponding author.
